# P-2074. Prevalence and health care utilization outcomes of Z codes in patients with HIV, Tuberculosis Disease, Hepatitis B Virus Infection, and Hepatitis C virus Infection

**DOI:** 10.1093/ofid/ofaf695.2238

**Published:** 2026-01-11

**Authors:** Jenna M Wick, C William Pike, Jananee Muralidharan, Gavin Hui, Daisuke Furukawa

**Affiliations:** Stanford University, Palo Alto, CA; Atropos Health,, New York, New York; Atropos Health,, New York, New York; Atropos Health, New York, New York; Stanford University, Palo Alto, CA

## Abstract

**Background:**

There is growing recognition that social determinants of health (SDH) play a larger role than medical care. One method to identify SDH is with International Classification of Diseases, Tenth Revision (ICD-10) diagnosis codes known as Z codes, but the prevalence and utility of Z codes have not been studied in infectious diseases.

**Figure:**
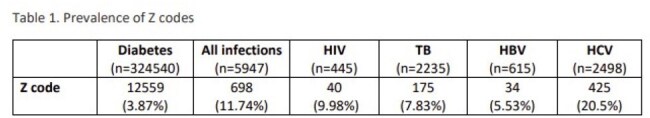

**Figure:**
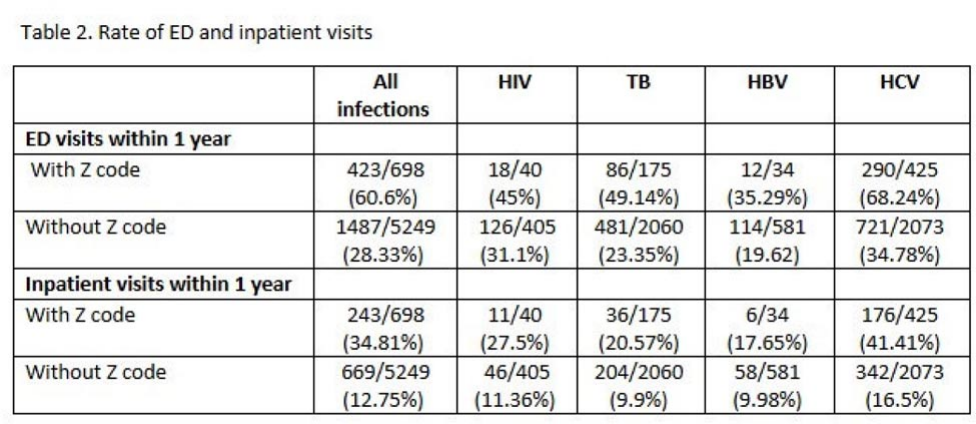

**Methods:**

This retrospective cohort study used the “Apollo” Dataset on the Atropos Evidence Network, a national dataset with EHR data and linked claims from 2015-2023. We found Z code (ICD-10 codes Z55-65) prevalence in adults with a first ICD-10 HIV, tuberculosis (TB), hepatitis B (HBV) or hepatitis C (HCV) diagnosis, compared to those with a first diabetes diagnosis. Patients with multiple inclusion diagnoses were excluded. Among patients with infections, we compared the rates of ED and inpatient visits within 1 year in those with Z codes versus those without. Propensity scoring was used to balance confounders.

**Results:**

5,947 patients had infections (445 HIV, 2235 TB, 615 HBV, 2498 HCV) and 324,540 patients had diabetes. Z code prevalence was higher in patients with infections (n=698, 11.74%) compared to patients with diabetes (n=12559, 3.87%) (p< 0.001), and this trend was consistent across infection types (Table 1). After propensity score matching, patients with these infections were more likely to have a Z code (OR 1.74 p < 0.001) than patients with diabetes. Among patients with infections, those with Z codes had a higher rate of ED visits (60.6% vs 28.33%, p< 0.01) and inpatient visits (34.81% vs 12.75%, p< 0.001) compared to those without, and this was observed across infection types (Table 2). After propensity score matching, those with a Z code were more likely to have an ED visit (OR 1.91, p < 0.001) and inpatient visit (OR 1.9, p < 0.001) compared to those without.

**Conclusion:**

This large national cohort study found patients with HIV, TB, HBV and HCV had a higher prevalence of Z codes than patients with diabetes. Among patients with these infections, those with Z codes required significantly higher healthcare utilization. All medical conditions are an issue of health equity, but it is acutely apparent in infectious diseases. Determining patient populations with high social needs may identify areas for interventions to improve health outcomes.

**Disclosures:**

All Authors: No reported disclosures

